# Late Response of Antiretroviral Therapy in an HIV-1-Infected Patient due to Hepatitis B and C Coinfections: The First Case Report in Nepal

**DOI:** 10.1155/2019/2070973

**Published:** 2019-02-11

**Authors:** Sundar Khadka, Rupendra Shrestha, Sanjeet Pandit, Roshan Pandit, Anup Bastola

**Affiliations:** ^1^HIV Reference Unit, National Public Health Laboratory (NPHL), Teku, Kathmandu, Nepal; ^2^Dr. Koirala Research Institute for Biotechnology and Biodiversity, Kathmandu, Nepal; ^3^Sukraraj Tropical & Infectious Disease Hospital, Teku, Kathmandu, Nepal

## Abstract

**Aim:**

Dual coinfection of HCV and HBV in HIV-1-infected population is a leading cause of morbidity and mortality. Also, they share routes of HIV transmission; however, it might be associated with an independent factor like injecting drug use for HCV and unsafe sex for HBV. This case report suggests that hepatitis virus coinfection may lead to late response of antiretroviral therapy (ART) in HIV-1 patients.

**Patients and Methods:**

A 49-year-old male patient visited for the routine follow-up investigation at the National Public Health Laboratory (NPHL), Teku, Nepal. He was an HIV-1-positive injecting drug user (IDU) co-infected with HCV and HBV. The patient was under ART as per the National HIV Testing and Treatment Guidelines 2017, Nepal. Further, serological and viral load testing was performed for confirmation and monitoring therapy, respectively.

**Results:**

It is the first report that highlights the dual coinfection of HCV and HBV in an HIV-1 patient from Nepal. The follow-up investigation shows improved response to ART with an increase in CD4+ cells. However, detectable viral loads indicated for a late response might be due to effects of coinfections or viral interactions.

**Conclusions:**

Dual coinfection is rare; however, it is more serious with poorly defined epidemiology and evolution in an HIV-1-infected population. Thus, universal screening of HBV or/and HCV coinfection in HIV-1-infected population requires immediate implementation for true prevalence, proper management, and early intervention.

## 1. Introduction

Hepatitis B virus (HBV), hepatitis C virus (HCV), and human immunodeficiency virus (HIV) are common viral infections that share routes of transmission through unprotected sex and exchanging needles/syringes. HBV and HCV coinfections are widespread among HIV-infected patients worldwide, which cause long-term illness to chronic hepatitis and death. Viral hepatitis progresses faster in HIV-infected patients compared to those without HIV. Although the antiretroviral therapy (ART) extended the life expectancy of people with HIV, viral hepatitis associated with HBV and HCV becomes the primary cause of morbidity and mortality. As documented, 36.7 million people are living with HIV/AIDS, and 1 million died of HIV-related illness worldwide in 2016. Among them, 3.5 million people are covered by Southeast Asia [1]. In Nepal, 30,646 people are HIV positive, and common risk groups were client of sex workers (34.1%), IDU (10.5%), migrant workers (9.3%), spouse migrants (6.3%), sex workers (4.9%), men having sex with men (1.8%), blood/blood products (0.4%), and others (32.8%) [2]. The estimated global prevalence of people living with chronic HBV and HCV infection was 240 and 184 million, respectively [3, 4]. The burden and clinical severity of HBV/HCV coinfection in HIV-infected people are rare but require continued follow-up with frequent testing of serum markers as well as molecular detection and quantification of viral nucleic acids with careful observation. The molecular testing of HBV and HCV coinfections in a key population of HIV patients is convincing to estimate the true prevalence that provides accurate and justifiable data. However, the data of HBV/HCV coinfection in Nepalese population are infrequently recognized, uneven quality, uncategorized, scattered, and rarely reported.

In this case report, we present a rare case of HBV and HCV coinfections in an HIV-1-infected patient with an improved CD4+ count but detectable viral loads after ART. Such triple coinfections in the patient from developing countries like Nepal provide an opportunity to access the effects of viral hepatitis coinfection on immediate and long-standing outcomes after antiretroviral therapy. Also, coinfection could be an exciting model for viral interaction studies and their clearance in response to immune cells.

## 2. Case Presentation

### 2.1. Patient and Method

The patient was a 49-year-old Nepalese man who was an HIV-1-positive injecting drug user coinfected with hepatitis B and C. He was informed and written consent was obtained for collection of blood samples for follow-up investigation. All the necessary tests and analysis were performed at the National Public Health Laboratory (NPHL), Kathmandu, Nepal. The blood sample was collected in a plain and K2 EDTA tube (BD Vacutainer). The rapid diagnostic testing for HIV-1/2, HBV, and HCV was performed using rapid immunochromatography, while syphilis testing was done using the flocculation method for VDRL (RPR). The reactive and nonreactive results were further confirmed by enzyme-linked immunosorbent assay for HBsAg, anti-HCV, and anti-HIV 1/2 (ELISA Human, Germany) and electrochemiluminescence immunoassay for HBeAg, HBsAg, anti-HBs, anti-HBe, anti-HBc, anti-HCV, and HIV Combi PT (ECLIA, cobas Roche Inc., Germany). The ECLIA was performed using cobas e 411 analyzer (Roche Inc., Germany). The whole blood collected in EDTA was used for CD4+ count using a BD fluorescent-activated cell sorter system (BD Biosciences, San Jose, CA, USA). The viral nucleic acid (DNA/RNA) was extracted using the QIAamp® DSP Virus kit (Qiagen, Germany). HBV DNA and HCV RNA were quantified by Corbett Rotor-Gene 6000 Real-Time PCR. The Artus HBV/HCV RG PCR kit (Qiagen, Germany) allows for a viral load detection limit of 10–100,000,000 IU/ml for HBV-DNA and 65–1,000,000 IU/ml for HCV-RNA with 97% specificity. HIV-1 was amplified and quantified by a Cobas® TaqMan® 48 analyzer. The COBAS® AmpliPrep/COBAS® TaqMan® HIV-1 Test, v2.0 (Roche Molecular Systems, Inc., USA), has an assay of linearity from 20 to 10,000,000 copies/ml with 100% specificity.

### 2.2. Follow-Up Investigation and Laboratory Findings

**Figure 1:**
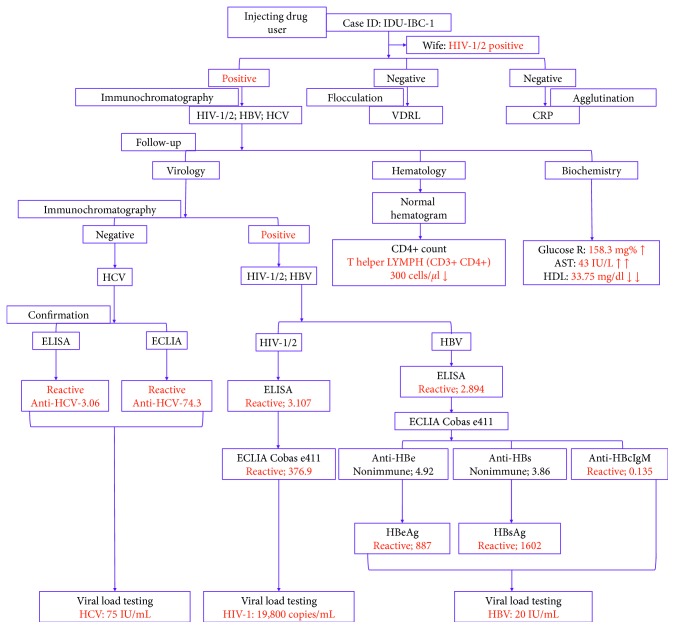


The patient was tested positive for HIV-1/2 infection with hepatitis B and C coinfections first time at a tertiary care hospital of Nepal on 14 August 2017. The initial finding at the time of HIV-1/2 confirmation showed decreased CD4+ nadir and SGOT. The client was informed that he was HIV-1/2 positive, and appropriate counseling was provided regarding the HIV, risk factor, transmission to family, social issues, and availability of ART. He was prescribed a fixed-dose combination of tenofovir disoproxil fumarate (TDF), lamivudine (3TC), and efavirenz (EFV) as the preferred option to initiate ART according to the National HIV Testing and Treatment Guidelines 2017, Nepal. It also covers for HBV infection. However, the patient was not in HCV treatment. He also mentioned that his spouse was positive for HIV-1/2. After ten months of ART initiation, he visited for follow-up on 5 June 2018. The follow-up investigation includes a routine laboratory test on hematological parameters, biochemical parameters, and viral loads. The HCV was tested negative using rapid chromatography but confirmed positive using ELISA and ECLIA. However, the improved CD4+ count was observed after ART. Final testing of viral loads for HIV-1, HBV, and HCV was performed for monitoring of ART. The schematic flowchart of the case presentation is shown in Figure 1, and laboratory investigation of patient test reports is presented in Table 1.

## 3. Discussion

Hepatitis B and C co-primary infections in the HIV-infected population have inconsistent prevalence among the reported studies and countries. The primary determinant for such dual coinfections in key HIV-infected population might be associated with routes of HIV transmission. Even HBV, HCV, and HIV share routes of transmission, but coinfection with viral hepatitis has independent predictors; that is, HCV infection is more closely linked to injection drug use and HBV is associated with unsafe sexual intercourse. In comparison to females, the males showed a significant association of HBV and HCV dual coinfection among HIV-infected population [5]. Also, previous studies reported that the people aged between 30 and 40 or over 40 have a higher rate of hepatitis B and C coinfections in HIV-infected populations [6, 7]. A case with a similar association has been identified after retrospective analysis of laboratory findings and clinical history. A male patient, aged 49 and an injecting drug user, was reported as HIV positive with HBV and HCV coinfections. In the case described here, the follow-up investigation after ten months showed the negative results for HCV by rapid immunochromatography, but standard tests like ELISA and ECLIA showed positive for HCV. This indicates that the rapid diagnostic test might lead to false-negative results and thus enzyme-linked immunosorbent assay (ELISA) and electrochemiluminescence immunoassay (ECLIA) should be considered as the standard tests for screening viral hepatitis coinfection in HIV [8, 9]. The abnormality in liver functions and changes in the liver enzymes as an effect of antiretroviral therapy or hepatitis coinfection might cause serious complication in the patients. The liver function test revealed the elevation of aspartate transaminase (AST) in the patient serum as hepatitis coinfection in the HIV-1-infected patient develops a higher risk for hepatic enzymes elevation on ART [10]. In contrast to elevated AST, the APRI (AST to platelet ratio index) score of 0.61 indicates that the patient does not have liver cirrhosis due to HBV and HCV coinfections. Besides, the improvement in the CD4+ count as compared to initial findings was observed after the ART. But still, a lower CD4 count after ten months of ART initiation might be associated with HBV/HCV coinfections because poor CD4 count responses were observed in HCV coinfection among HIV-infected patients [7]. Moreover, poor CD4 responses depend either on the increased replication of viral genome or immune status of the patient as well. Despite the improved CD4+ count from 89 to 300 cells/*µ*L after ART, the laboratory finding showed detectable copies of HIV genome including HBV and HCV. It might be due to the late response of ART or effects of coinfections and/or viral interactions. Thus, measuring the viral load becomes crucial for monitoring of therapy and follow-up investigation of viral replication status as well as clearance. Furthermore, the true prevalence of HBV and HCV dual infection in HIV could be more in detecting the viral nucleic acids. The uncategorized and scattered data are not sufficient to indicate the true prevalence of HBV/HCV dual infections in the HIV population based only on serological assessment. The few seroprevalence studies in Nepal also revealed that the higher prevalence of HBV is associated with blood donors and sex workers while HCV infection is related to injection drug use.

In Nepal, the seroprevalence of HBV and HCV infections in blood donors nationwide is 0.82% and 0.47%, respectively, while at central blood transfusion center (CTBS) in Kathmandu, the seroprevalence was 0.92% and 0.71%, respectively [11]. But, in people who inject drugs, the seroprevalence rate for HBV (HBsAg), HCV (anti-HCV), and HIV was 3.5%, 49.9%, and 13.8%, respectively [12]. However, the HBV and HCV coinfections in HIV-infected patients were 3.2% and 4.1%, respectively, but no HBV/HCV coinfection were reported from western Nepal [13]. Also, there was 9.1% of HBV coinfection in HIV-infected female sex workers or sex-trafficked women/girls [14]. Also, 7.1% of HBV, 18.1% of HCV, and 0.5% of HBV/HCV infection living with HIV were observed in people who inject drugs [12]. The first nationally representative cross-sectional study on people living with HIV and female sex workers revealed 4.4% of HBV, 19% of HCV, and 1% of HBV/HCV coinfection [15]. However, the prevalence of HCV could be higher than that of HBV coinfection in HIV-infected patients due to the availability of HBV vaccines. Still, in comparison to HCV, sexual transmission of HBV is higher and more often in IDU. The variance prevalence rate of viral hepatitis coinfection in key HIV-infected population associated with the studied group, age factor, transmission risk behavior of the group, increased loss of follow-up investigation, standard screening and confirmation technique, effective intervention program, and availability of proper therapy. Dual coinfection of HBV/HCV could be more serious in comparison to a patient with either HBV or HCV. The management of liver cirrhosis associated with hepatitis virus that causes rapid progression is not considered serious. There is a lack of a surveillance system to monitor viral hepatitis, a lack of awareness program about coinfection, and neither prevention strategies nor intervention program to provide training/skills for avoiding infection levels in HIV patients. The most effective standard needle and syringe exchange programs should be conducted to minimize the rate of hepatitis virus coinfection in HIV patients. Moreover, the prevention of coinfection has been of interest to public health authorities in Nepal. We urged health professionals and government health authorities to implement standard routine screening methods for hepatitis B and C infections in all HIV-positive patients for proper management and early intervention.

## 4. Conclusions

In conclusion, triple infections are rare cases neither investigated nor reported from Nepal. Our case of triple infections reveals good immune response and no cirrhosis due to high-level adherence (>95%) to ART. However, the detectable viral loads might be due to the influence of late response, effects of coinfections, and/or viral interactions in HIV-1 genome. Therefore, current prevention strategies on the parenteral transmission of HBV and HCV should be revised to alert the importance of using standard needles, avoiding sharing equipment, and unprotected sexual intercourse that could be based on family and community.

## Figures and Tables

**Table 1:** Laboratory investigation of HBV/HCV coinfection in an HIV-1-infected patient after ten months of antiretroviral therapy.

*Tests*	*Method*	*Results*	
*Rapid diagnostic tests*			
HIV-1/2	Immunochromatography	Positive	
HBsAg	Immunochromatography	Positive	
Anti-HCV Ab (IgM)	Immunochromatography	Negative	

*Tests*	*Result*	*Reference ranges*	
*Biochemical parameters*			
Glucose R	158.27↑	70–150 mg%	
ALP	85	44–147 IU/L	
SGPT/ALT	36	7–57 IU/L	
SGOT/AST	43↑↑	37 IU/L	
HDL	33.750↓↓	>50 mg/dl	

*Hematological parameters*			
Complete blood count (CBC) is in normal limit			

*CD4+ count*			
Percentage of T lymphocytes (CD3+ and CD45+)	75	55–84%	
Absolute count of T lymphocytes (CD3+)	1160	690–2540 cells/*µ*L	
Percentage of T-helper lymphocytes (CD3+ CD4+/CD45+)	15	31–60%	
Absolute count of T-helper lymphocytes (CD3+ CD4+)	300↓↓	410–1590 cells/*µ*L	
Absolute count of lymphocytes (CD45+)	1551 cells/*µ*L		

*Tests*	*Results*	*Cutoff index*	*Interpretation*
*Enzyme-linked immunosorbent assay(ELISA)*			
HBsAg	2.894	COI: 0.15, reactive: >0.15, nonreactive: <0.15	Reactive
HCV	3.067	COI: 0.165, reactive: >0.165, nonreactive: <0.165	Reactive
HIV-1/2	3.107	COI: 0.131, reactive: >0.131, nonreactive: <0.131	Reactive

*Electrochemiluminescence immunoassay(ECLIA)*			
HBeAg	887	>1.0 reactive, <1.0 nonreactive, boarder line 0.9-<1.0	Reactive
HbsAg	1602	>1.0 reactive, <1.0 nonreactive, boarder line 0.9-<1.0	Reactive
Anti-HBs	3.86	<10.0 nonimmune, >10.0 immune	Nonimmune
Anti-HBe	4.92	<1.0 reactive, >1.0 nonreactive	Nonreactive
Anti-HBcIgM	0.135	<1.0 reactive, >1.0 nonreactive	Reactive
Anti-HCV	74.3	>1.0 reactive, <1.0 nonreactive, boarder line 0.9-<1.0	Reactive
HIV-COMBI PT	376.9	>1.0 reactive, <1.0 nonreactive, boarder line 0.9-<1.0	Reactive

*Tests*	*Results*	*Assay range*	*Interpretation*
*Quantitative PCR*			
HBV DNA	20 IU/ml	10–100,000,000 IU/ml	Detected
HCV RNA	75 IU/ml	65–1,000,000 IU/ml	Detected
HIV-1 RNA	19,800 copies/ml	20–10,000,000 copies/ml	Detected

## Additional Points

(i) We report the first evidence of HCV and HBV coinfections in an HIV-1 patient who revealed late response of ART which might be due to viral nucleic acid interaction. (ii) Hepatitis virus coinfection in HIV-infected patients is common, but lack of systemic and categorized studies leads to a low prevalence rate. (iii) Detection and quantification of plasma viral nucleic acid should be considered for true prevalence and also for monitoring therapy in the long-term follow-up investigation. (iv) Thus, the impact and evolution of viral hepatitis in HIV-viral response to ART remain unclear and matter of research.
